# Single-Layer Full-Color Waveguide Display Based on a Broadband Efficient Meta-Grating

**DOI:** 10.3390/nano15191493

**Published:** 2025-09-30

**Authors:** Yong Li, Fei Wu, Huihui Li, Mengguang Wang, Zhiyuan Xiang, Zhenrong Zheng

**Affiliations:** 1 College of Optical Science and Engineering, Zhejiang University, Hangzhou 310027, China; liyong@llvision.com (Y.L.);; 2 Beijing LLVision Technology Co., Ltd., Beijing 100176, China

**Keywords:** augmented reality, metaverse, meta-grating, optical waveguide, AR displays

## Abstract

Augmented reality (AR) displays are pivotal for delivering immersive experiences in the metaverse, thus driving significant research interest. Current AR systems, predominantly relying on diffraction principles, often suffer from low efficiency and face challenges in realizing monolithic full-color operation. Herein, we propose an AR system that integrates a broadband and highly efficient meta-grating in-coupler and an elliptical meta-grating out-coupler onto a single thin glass substrate. The meta-gratings, with unique nanostructures, enable coupling efficiency exceeding 60% for red (R), green (G), and blue (B) wavelengths across the entire field of view (FOV). Image-bearing light is first coupled into a single-layer optical waveguide via the meta-grating, then undergoes two-dimensional expansion through the elliptical meta-grating, and is ultimately coupled into the human eye to form a large AR FOV. Experimentally, we fabricated an optical waveguide prototype and validated the system’s high efficiency and color-enhanced imaging capabilities. This work advances the development of monolithic, trichromatic, highly efficient, and large FOV AR displays based on meta-grating technology.

## 1. Introduction

Augmented reality (AR) technology is a new technology that seamlessly integrates real-world information and virtual world information. Due to its ability to enhance the display output of the real environment, AR has more obvious advantages than virtual reality display technology in fields such as medical research [1], precision instrument manufacturing and maintenance [2,3], military aircraft navigation [4], engineering design [5], and remote robot control [6,7]. As a new human–computer interface and simulation tool, AR has been developing rapidly and has attracted considerable attention over the past decade. The display systems used in AR glasses currently on the market are combinations of various miniature displays and optical components [8,9] such as prisms, freeform surfaces, Birdbaths, and optical waveguides.

Among the above optical imaging components, optical waveguide technology is a more distinctive optical component developed in response to the needs of AR [10,11]. Due to its light weight and high transmittance for external light, it is regarded as an essential optical solution for consumer-grade AR glasses. With the adoption and mass production of optical waveguides in Microsoft’s two generations of HoloLens products and Magic Leap One, discussions about optical waveguides have also increased [12,13]. Among them, diffractive optical waveguides are currently the most promising direction due to their large field of view (FOV) and compatibility with semiconductor processing technologies [12,14]. However, the physical process of diffraction itself has selectivity for angles and wavelengths, leading to dispersion issues. Ordinary gratings struggle to balance the range of color bands and incident angles they cover [15,16]. How to use a single layer of grating to act on the three RGB colors while achieving the maximum FOV is a challenge in the industry. In addition, the low efficiency of coupled gratings also places high demands on the brightness of the display. In recent years, the metasurface proposed by Professor Capasso has attracted significant attention [17,18]. Compared with optical elements based on refraction and diffraction—such as lenses, polarizers, filters, and absorbers—dielectric metasurfaces utilize the Mie scattering principle to manipulate electromagnetic waves, enabling flexible control over the direction and efficiency of light [19,20]. Therefore, metasurfaces are expected to solve a series of problems associated with diffractive optical waveguides [21,22,23,24].

We designed a meta-grating waveguide based on a dielectric waveguide phase shifter for the visible light band. This waveguide leverages a precisely controlled phase gradient to achieve a uniform first-order diffraction coupling efficiency exceeding 60% for red (R), green (G), and blue (B) wavelengths across the entire FOV. Specifically, light encoded with color image information is initially coupled into the optical waveguide system through an in-coupling meta-grating, which consists of two nanobeams with distinct widths. Subsequently, two-dimensional beam expansion is accomplished via an out-coupling meta-grating fabricated on a fused-silica substrate, and the expanded image is finally coupled into the human eye for visualization. As a prototype demonstration, the projection of clear RGB images not only confirms the high efficiency of the entire system but also validates the feasibility of color imaging based on a single-layer waveguide integrated with the designed silicon nitride (*Si*_3_*N*_4_)-based meta-gratings.

## 2. Design of the Meta-Grating In-Coupler

**Figure 1:**
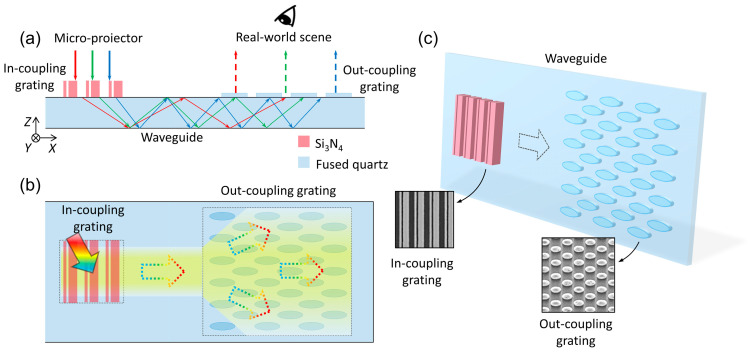
Schematics of a dielectric waveguide-based meta grating-waveguide. (**a**) Side view: Light from a micro-projector couple in via an in-coupling grating, propagates through total internal reflection in the *Si*_3_*N*_4_-glass waveguide, and couples out for viewing with the real-world scene. Here, the red, green, and blue arrows in the figure represent visible light, and the same arrows used elsewhere in the text carry the same meaning. (**b**) Top view: Depicts light coupling and propagation via the in-and out-coupling gratings. (**c**) Grating structures: Illustrates the evolution of in-and out-coupling gratings into nanostructured forms for light manipulation.

Figure 1 presents the schematic diagram of the proposed diffractive waveguide display based on a dielectric waveguide phase shifter. The system is composed of a micro-display and a glass waveguide, where the glass waveguide is integrated with a metasurface grating in-coupler and an elliptical metasurface grating out-coupler. Specifically, Figure 1b,c show the detailed design, while Figure 1a further presents the system demonstration. The phase-shifting grating designed based on the dielectric waveguide phase shifter—i.e., the metasurface grating in-coupler—achieves uniform coupling efficiency at R, G, B wavelengths by virtue of the phase gradient of the metasurface. The specific process of light propagation and modulation is as follows: the linearly polarized image-carrying light from the micro-display is first deflected by the metasurface grating along the direction of the phase gradient; after deflection, the angle at which the light tilts toward the bottom surface of the waveguide are larger than the critical angle for total internal reflection, which ensures that total internal reflection occurs when the light propagates in the waveguide. In most regions of the waveguide, the light undergoes multiple diffractions when passing through the elliptical metasurface grating, thereby realizing two-dimensional beam expansion and coupling the image into the human eye. The periodic design of the in-coupler and out-coupler must ensure vector closure to prevent distortion of the projected image after it travels along the folded optical path in the waveguide.

**Figure 2:**
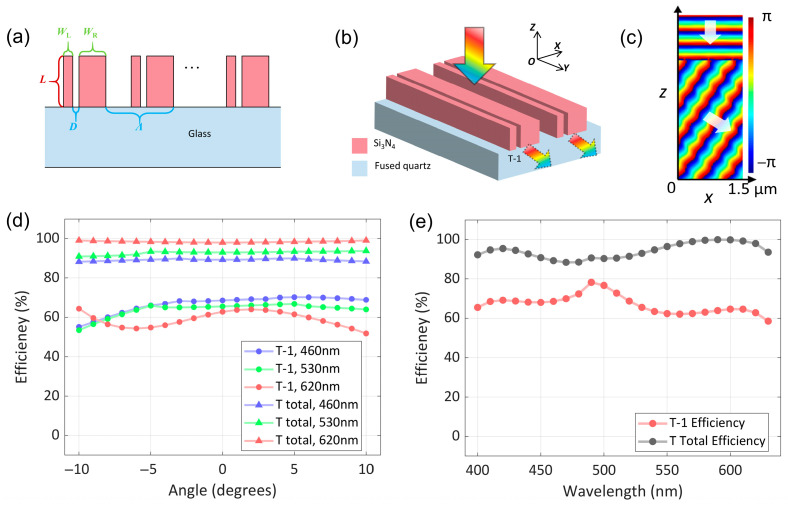
Schematics and performance of a *Si_3_N_4_* meta-grating for in-coupling. (**a**) Schematic of the meta-grating structure on a glass substrate, with nano-pillars of varying widths. (**b**) 3D view illustrating light coupling into the meta-grating and propagation. (**c**) Simulated phase distribution in the *xz*-plane, showing phase difference between nano-pillars. (**d**) Simulated efficiency of the meta-grating versus incident angle at wavelengths 460 nm, 530 nm, and 620 nm. (**e**) Simulated efficiency of the meta-grating versus wavelength, with data for first-order (T-1) and total (T total) diffraction efficiencies.

The design of the in-coupler grating is a critical part of the optical waveguide design, as it not only determines the diffraction efficiency of the grating but also affects the uniformity of the waveguide. Traditional blazed gratings and slanted gratings used for incident coupling struggle to balance the diffraction efficiency of RGB wavelengths and the FOV. Therefore, to achieve full-color imaging, most waveguide-based AR glasses adopt a structure containing two or three gratings on different layers, with each grating dedicated to a specific wavelength. Figure 2a shows the proposed *Si*_3_*N*_4_ meta-grating—a phase-shifting element. Its propagation direction is along the -*z* axis, and light can be coupled from air into glass through this grating. By adjusting the widths of the two nano-pillars of the grating, the effective refractive index of the fundamental mode can vary over a wide range: when light propagates mainly in *Si*_3_*N*_4_, the effective refractive index *n_eff_* ≈ *n_Si__3__N__4_*; when light propagates mainly in air, the effective refractive index *n_eff_* ≈ *n_air_*. If two nano-pillars are placed side by side and the optical coupling between them is negligible, the phase shift Δ*φ* accumulated by the light when propagating along the entire meta-grating will be proportional to the grating length *L*:

(1) $$\Delta \varphi =\frac{2\pi }{\lambda }\Delta n_{eff}L$$

where Δ*n_eff_* is the effective index difference between the two nano-pillars. A phase difference of Δ*φ* = 2*π* can be obtained over a subwavelength propagation length of

(2) $$\Delta n_{eff}=\frac{\lambda }{L}$$

We first consider the simple case involving only a meta-grating within an aperture. By placing two nano-pillars with a phase difference of Δ*φ* at a subwavelength distance *D*, one can direct the beam to an angle *θ*. The value of *θ* depends on Δ*φ*, *λ*, and *D*, and is governed by the condition for constructive interference along the direction defined by *θ*:

(3) $$\Delta \varphi =\frac{3\pi D}{2\lambda }\sin(\theta )$$

The relationship between the grating period Λ, angle *θ*, and the refractive index of the glass *n* can be derived by the formula:

(4) $$\sin(\theta )=\frac{\lambda }{n\Lambda }$$

Figure 2a shows a schematic of the meta-grating structure on a glass substrate, which consists of nano-pillars with different widths. Figure 2b illustrates the generation of phase difference between waveguides with different widths, while Figure 2c simulates the wavefront of transmitted light under various polarization states. These results confirm that, under the action of the meta grating, the energy of polarized light is concentrated in the first diffraction (T-1-order). To achieve efficient transmission of RGB light, the meta-grating was designed via finite difference time domain (FDTD) scanning and formula calculations. It comprises two nano-pillars with the same length (*L* = 500 nm) but different widths (*W_L_* = 55 nm and *W_R_* = 200 nm), separated by a slit of *D* = 50 nm, and its period is determined by the deflection angle (∧=487 nm). Subsequently, we simulated the wavelength-dependent absolute efficiency of the *Si_3_N_4_*-based meta-grating (the absolute efficiency is defined as the ratio of the transmitted power in different diffraction orders to the input power), with the results shown in Figure 2d. Across the entire visible spectrum, the first-order diffraction efficiency of the *Si*_3_*N*_4_-based meta-grating basically exceeds 60%. Figure 2e presents the T-1-order diffraction efficiency of the meta-grating at a wavelength range of 400~700 nm, confirming its suitability for wide-wavelength applications.

## 3. Design of the Grating Out-Coupler

**Figure 3:**
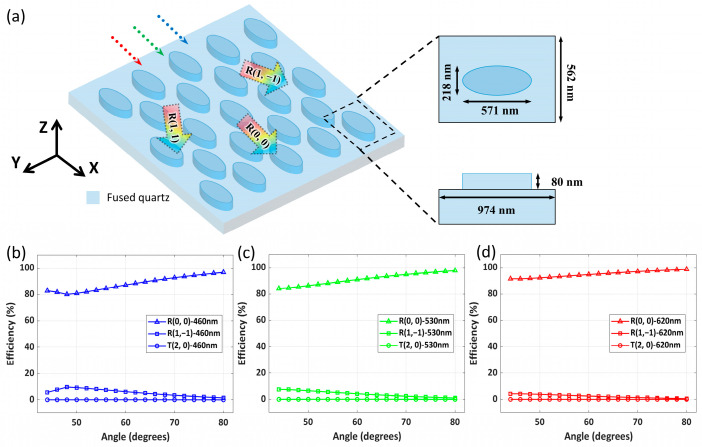
Schematics and diffraction efficiency of an elliptical meta-grating for out-coupling. (**a**) 3D schematic of the elliptical meta-grating on a glass substrate, with inset showing unit cell dimensions. (**b**–**d**) Simulated absolute diffraction efficiencies versus incident angle (40–80°) at wavelengths (**b**) 460 nm, (**c**) 530 nm, and (**d**) 620 nm, for specified diffraction orders.

The out-coupling grating has a special two-dimensional elliptical structure, as depicted in Figure 3a. Essentially, it is simplified from two one-dimensional gratings with a 60° angle, enabling simultaneous two-dimensional beam expansion and image coupling into the human eye. When total reflection light arriving along the *x*-axis impinges on the coupling grating, multiple diffractions occur via interaction with the elliptical meta-grating. Light can be diffracted into the zero-order R (0, 0), which accounts for the majority and sustains the propagation of incident light. It can also be diffracted into the second-order through the elliptical meta-grating; this second-order waveguide light is coupled along the positive *z*-axis toward the observer and designated as T (2, 0). As shown in Figure 3b–d, the diffraction efficiencies of T (2, 0) at the three RGB operating wavelengths are presented when the incident angle varies across the full FOV, 40° to 80°. Additionally, light can be diffracted into the first order through the elliptical optical structure. This order is diffracted at 60° or −60° relative to the *x*-axis and further interacts with other elliptical meta-gratings, defined as R (1, 1) and R (1, −1). Collectively, these diffraction behaviors allow the elliptical out-coupling grating to efficiently regulate light propagation for display applications.

## 4. Experiments and Results

**Figure 4:**
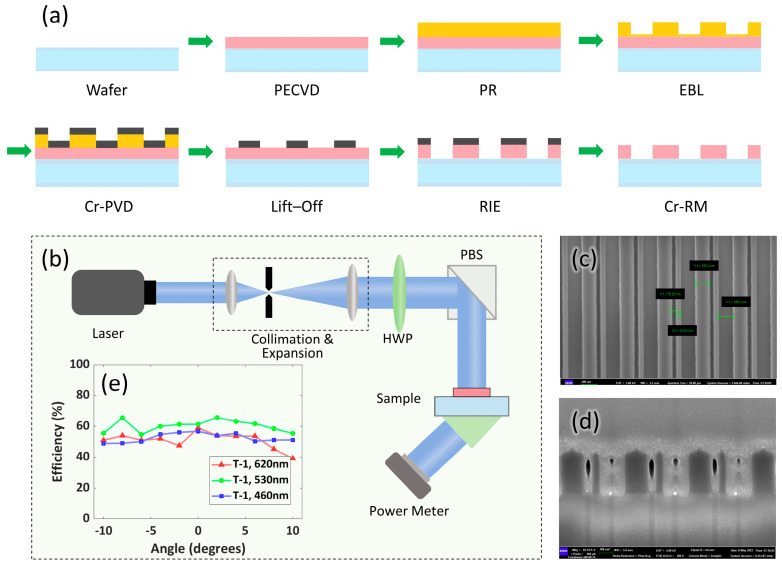
Fabrication, characterization, and performance of meta-gratings. (**a**) Fabrication process flow for meta-gratings, involving PECVD, EBL, RIE, etc. (**b**) Experimental setup for measuring in-coupling efficiency, with laser, collimation/expansion, and power meter. (**c**,**d**) SEM images of meta-gratings (scale bar: 500 nm). (**e**) Measured absolute first-order diffraction efficiency of the meta-grating across visible wavelengths (460 nm, 530 nm, 620 nm) versus incident angle.

To verify the grating performance, we fabricated a meta-grating made of *Si*_3_*N*_4_, with a size of 3 mm × 3 mm, on a 0.7 mm-thick fused glass waveguide. Figure 4a illustrates the fabrication process flow of the meta-grating, encompassing steps like wafer preparation, plasma-enhanced chemical vapor deposition (PECVD), photoresist (PR) application, electron beam lithography (EBL) patterning, chromium physical vapor deposition (Cr-PVD), lift-off, reactive ion etching (RIE), and chromium removal (Cr-RM). These figures collectively provide clear insights into the meta-gratings’ fabrication process and experimental measurement basis.

Figure 4b presents the complete experimental setup for measuring the first-order diffraction efficiency of the meta-grating, following a sequential optical path: light is first emitted from a laser, then passes through collimation and expansion optics to form a uniform parallel beam. This beam subsequently travels through a half-wave plate (HWP) for polarization adjustment, and then is directed by a polarizing beam splitter (PBS) onto the meta-grating sample. Finally, a power meter is used to measure the transmitted light and quantify the first-order diffraction efficiency. Figure 4c is the scanning electron microscope (SEM) image of the meta-grating, showcasing its detailed microstructure. Figure 4d is the SEM image of the elliptical meta-grating, displaying its distinctive elliptical microstructure. For characterizing the meta-grating, we measured its first-order diffraction efficiency using a continuously tunable color laser FISBA READY Beam and THORLABS PM400 Optical Power Meter. RGB wavelength is individually controllable for precise performance. TE-polarized light was incident on the meta-grating, and the T-1 light was coupled into the fused silica waveguide. Figure 4e depicts the experimental results, which match well with the simulation results. The sample achieved an absolute efficiency above 40% in the visible band for wavelengths 460 nm, 530 nm, and 620 nm. This confirms the meta-grating’s effective performance in visible light.

**Figure 5:**
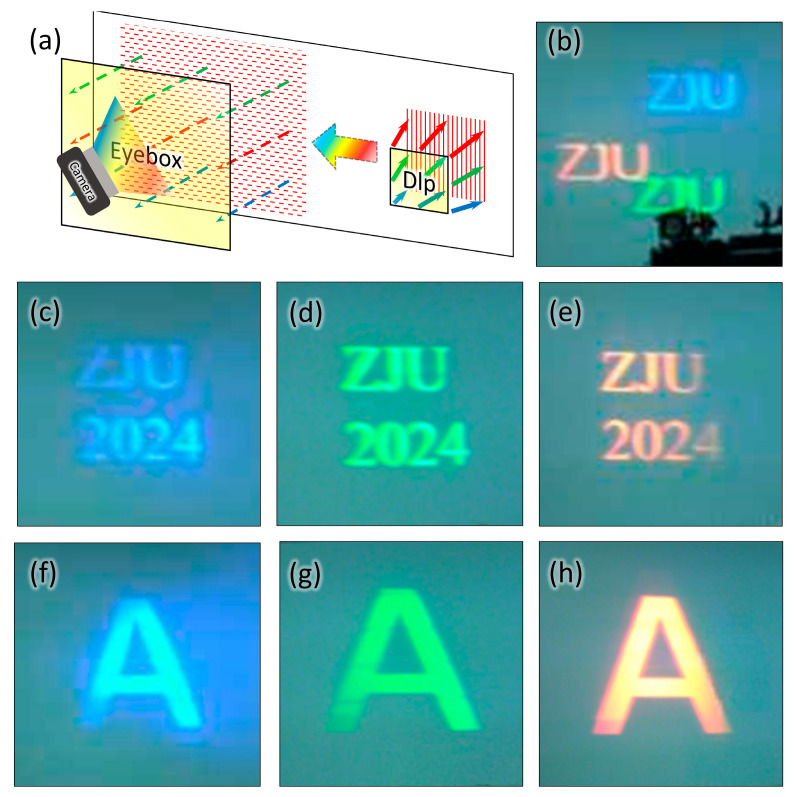
Demonstration of color virtual image display via a meta-grating-based stereoscopic waveguide. (**a**) Schematic of color virtual images (with eye-box) overlapping an ambient scene, received by a camera. (**b**) Captured image of “ZJU” color virtual images overlaid on an ambient scene. (**c**–**e**) Captured “ZJU 2024” images in blue color (**c**), green (**d**), and red (**e**). (**f**–**h**) Captured “A” images in blue (**f**), green (**g**), and red (**h**).

To test the color imaging effect of the optical waveguide system, we fabricated a meta-grating waveguide. The substrate is glass with a refractive index of 1.45, and the grating material is *Si*_3_*N*_4_. As illustrated in the working principle schematic in Figure 5a, a color image source is first input into a digital light processing (DLP) system. The output parallel light is then coupled into the meta-grating, and the image propagates forward through total reflection within the glass before being coupled to a camera via the elliptical meta-grating. The captured image in Figure 5b shows “ZJU” patterns in multiple colors overlaid on an ambient scene.

To further demonstrate the imaging performance of the meta-grating, separate imaging tests were conducted using different colors to verify the design feasibility. Specifically, Figure 5c presents the “ZJU 2024” image in blue color, Figure 5d,e display the “ZJU 2024” image in green and red color, and Figure 5f–h, respectively, show the letter “A” in blue, green, and red, collectively verifying the system’s capability to handle different color imaging tasks.

## 5. Conclusions

In this study, we propose and experimentally demonstrate an optical waveguide based on an all-dielectric phase shifter, incorporating a meta-grating in-coupler paired with an elliptical meta-grating out-coupler. By leveraging the phase gradient of the dielectric waveguide phase shifter, the meta-grating achieves a uniform coupling efficiency exceeding 40% across the visible light spectrum. The out-coupling grating, derived from the simplification of two orthogonal one-dimensional gratings arranged at a 60° angle, enables simultaneous two-dimensional beam expansion and efficient image coupling into the human eye. Finally, experimental demonstrations of color imaging confirm the feasibility and practical utility of the proposed system. Comprising merely two gratings, this AR waveguide display configuration significantly enhances system compactness, improves efficiency, and facilitates integration, while providing a broader exit pupil area. As an advanced AR display technology, this design holds substantial promise for widespread applications in the metaverse and related fields.

## Data Availability

No new data were created or analyzed in this study.

## Abbreviations

The following abbreviations are used in this manuscript:

AR	Augmented reality
FOV	Field of view
FDTD	Finite difference time domain
SEM	Scanning electron microscope
DLP	Digital Light Processing
PECVD	Plasma-enhanced chemical vapor deposition
PR	Photoresist
EBL	Electron beam lithography
Cr-PVD	Chromium physical vapor deposition
Cr-RM	Chromium removal
RIE	Reactive ion etching
HWP	Half-wave plate
PBS	Polarizing beam splitter
